# The capacity of action observation to drag the trainees' motor pattern toward the observed model

**DOI:** 10.1038/s41598-023-35664-w

**Published:** 2023-06-05

**Authors:** Maria Chiara Bazzini, Arturo Nuara, Giulio Branchini, Doriana De Marco, Laura Ferrari, Maria Chiara Lanini, Simone Paolini, Emilia Scalona, Pietro Avanzini, Maddalena Fabbri-Destro

**Affiliations:** 1 Consiglio Nazionale delle Ricerche, Istituto di Neuroscienze, Parma, Italy; 2 Dipartimento di Medicina e Chirurgia, Università degli Studi di Parma, Parma, Italy; 3 School of Advanced Studies, Università di Camerino, Camerino, Italy; 4 Dipartimento Specialità Medico-Chirurgiche, Scienze Radiologiche e Sanità Pubblica (DSMC), Università degli Studi di Brescia, Brescia, Italy; 5 Istituto Clinico Humanitas, Humanitas Clinical and Research Center, Milan, Italy

**Keywords:** Learning and memory, Motor control

## Abstract

Action Observation Training (AOT) promotes the acquisition of motor abilities. However, while the cortical modulations associated with the AOT efficacy are well known, few studies investigated the AOT peripheral neural correlates and whether their dynamics move towards the observed model during the training. We administered seventy-two participants (randomized into AOT and Control groups) with training for learning to grasp marbles with chopsticks. Execution practice was preceded by an observation session, in which AOT participants observed an expert performing the task, whereas controls observed landscape videos. Behavioral indices were measured, and three hand muscles' electromyographic (EMG) activity was recorded and compared with the expert. Behaviorally, both groups improved during the training, with AOT outperforming controls. The EMG trainee-model similarity also increased during the training, but only for the AOT group. When combining behavioral and EMG similarity findings, no global relationship emerged; however, behavioral improvements were "locally" predicted by the similarity gain in muscles and action phases more related to the specific motor act. These findings reveal that AOT plays a magnetic role in motor learning, attracting the trainee's motor pattern toward the observed model and paving the way for developing online monitoring tools and neurofeedback protocols.

## Introduction

Since early childhood, humans continuously learn new motor skills throughout all stages of life. For example, when people go for the first time to a sushi restaurant, their lack of motor experience induces them to observe other diners to figure out how to break, hold and use chopsticks and eat a nigiri. However, it is well known that observing others is not just a way to overcome the initial impasse. Still, it also represents a fundamental element promoting—via the mirror mechanism^1^—the acquisition of new motor skills^2^.

Recently, the reciprocal advantages of action observation and execution have been combined in the so-called Action Observation Training (AOT). Several studies proved the efficacy of AOT in facilitating the recovery of motor abilities in people with brain damage^2,3^, preventing corticomotor depression due to limb immobilization^4^, and limiting the subsequent decay of motor performance^5^. Beyond therapeutic and rehabilitative settings, AOT has been used for promoting the acquisition and refinement of new motor abilities^6–9^, with a major effect played by the regular alternation between action observation and execution^10^.

At the neural level, action observation can modulate corticospinal excitability^11–14^, induce a desynchronization of the mu rhythm^15–19^, and increase the metabolic consumption of fronto-parietal networks^20^.

The neural reactivity to action observation has been associated with the efficacy of AOT. Previous TMS studies demonstrated that the repeated administration of action observation induces neuroplastic changes larger than those due to the sole physical practice according to the congruence between the observed and executed actions^21^. Moreover, action observation combined with physical practice promotes the formation of motor memories in M1^22,23^. Neuroimaging studies suggested that motor skills improvement in patients undergoing AOT is associated with larger recruitment of motor brain regions, reflecting a reorganization of the motor circuits subserving the impaired functions^24–26^. The effect of AOT has also been demonstrated by Quadrelli and colleagues^27^, showing an increase in the mu rhythm desynchronization associated with motor improvement due to AOT in patients with cerebral palsy. Finally, a recent TMS study^28^ revealed that the corticospinal modulations induced by action observation might serve as predictors of the AOT outcome, further grounding the efficacy of AOT onto the mirror mechanism.

While most of the investigations to date assessed the cortical modulations associated with the AOT efficacy, few studies targeted the AOT impact on the peripheral boundaries of the motor system, *e.g.*, assessing how the temporal dynamics of muscular activation changes during the action observation training. Sparse findings investigated the electromyographical (EMG) modulations during action observation alone or combined with motor imagery/practice in tasks mainly involving force training^29–31^. Only one study^32^ has investigated the effects of AOT on EMG activity using a complex task requiring praxic organization (i.e., dart throwing). In this case, authors reported that training based on action observation reduced muscular contraction associated with behavioral improvement.

Even assuming that patterns of muscular activation change during AOT, it remains to be established whether the observed model can bias these changes. In other words, can the kinematics or electromyographic patterns of the trainee be *dragged* toward that of the model? If so, does this susceptibility set better premises for the AOT outcome? To address these issues, we designed a controlled EMG and behavioral study on 72 healthy participants to investigate the relationship between trainee-model motor *similarity* and the AOT outcome. A significant finding would shed light onto the neurophysiological mechanisms making action observation capable of conditioning the motor performance of the trainee during the learning of complex actions. In turn, such knowledge could guide the monitoring and online evaluation of training based on action observation.

## Materials and methods

### Participants

An a priori power analysis (within/between ANOVA) was conducted with G-Power 3.1 to define the sample size. The output showed a minimum sample size of 70 subjects (35 for each group) to obtain a significant effect on the dependent variable with an α = 0.05, power β = 0.90, and Cohen's F = 0.2.

Seventy-two healthy volunteers (age *M* = 26.03, *SD* = 4.25, range 19–40 years, 55 females) were enrolled in the experiment. All participants were right-handed according to the Edinburgh Handedness Inventory (Oldfield, 1971)^33^ (*M* = 0.82, SD = 0.17), had a normal or corrected-to-normal vision, and had no history of neurological or psychiatric disorder.

Participants were randomly subdivided into two groups: Action Observation Training (AOT, *n* = 36; age *M* = 26.08, *SD* = 4.25; 28 females and eight males) and Control (CTRL, *n* = 36; age *M* = 25.97, *SD* = 4.84; 27 females and nine males). The local ethics committee approved the study (Comitato Etico dell'Area Vasta Emilia Nord, n. 10,084, 12.03.2018), which was conducted according to the principles expressed in the Declaration of Helsinki. The participants provided written informed consent.

### Baseline evaluation

Participants were initially administered a questionnaire to evaluate their expertise with chopsticks; they were asked to rank on a Likert scale their chopsticks frequency use (scale = 1- less than once a year; 2- once or twice a year; 3- once or twice a month; 4- once a week; 5- more than once a week) and ability (scale = 1–6). Furthermore, the Nine Holes Peg Test (NHPT)^34^ was administered to evaluate the dominant and non-dominant hand dexterity.

### Stimuli and experimental design

**Figure 1:**
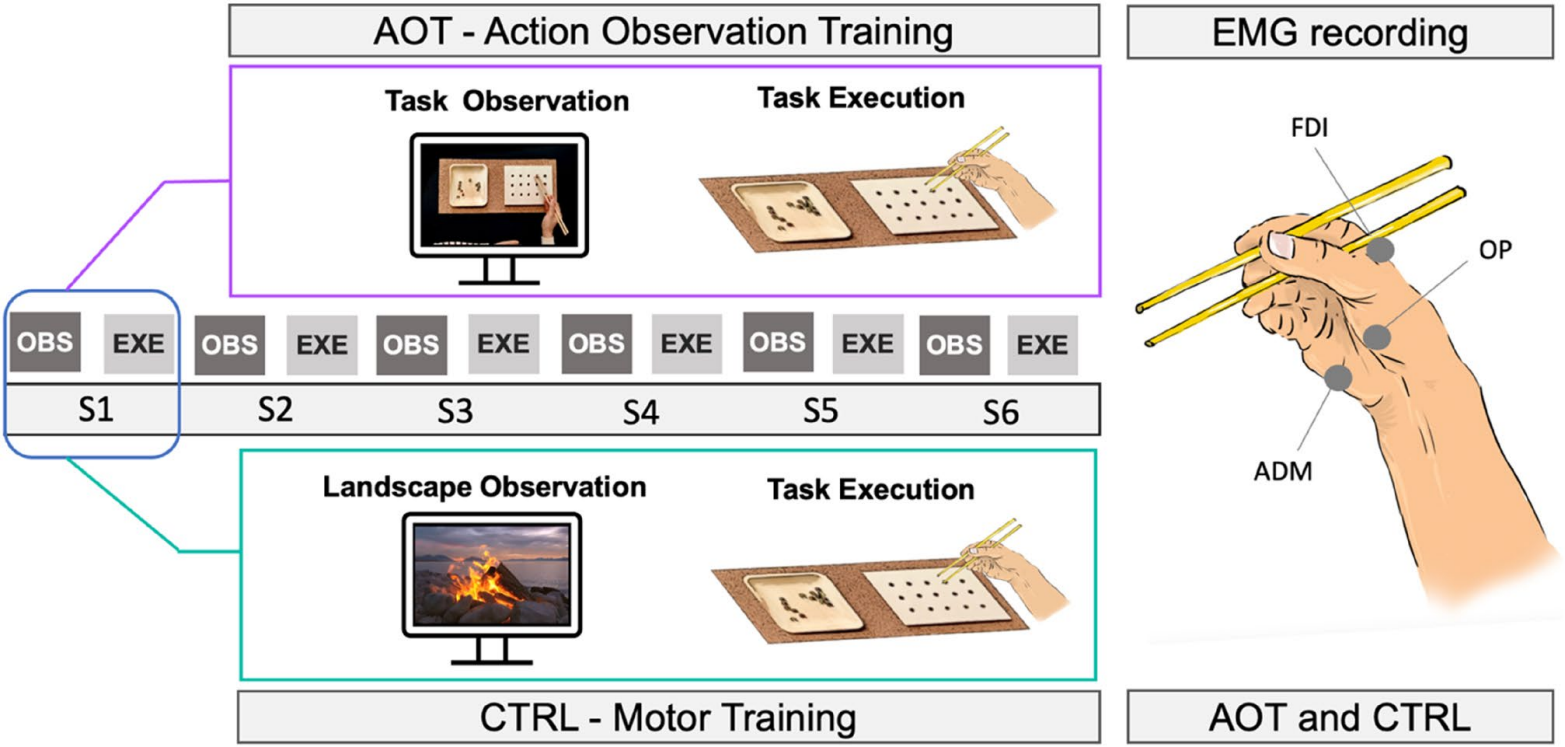
Experimental design. The two groups underwent training characterized by six sessions (S1-S6), each composed of an observation period and an execution one. The AOT participants observed a video of the expert performing the task and then executed the same task (violet panel). In contrast, the CTRL subjects observed a landscape video and then executed the task (green panel). EMG was recorded for both AOT and CTRL participants from three hand muscles: Opponens Pollicis (OP), First Digital Interosseous (FDI), and Abductor Digiti Minimi (ADM).

An expert native user of chopsticks was invited to perform the task of grasping with the chopsticks 15 marbles positioned on a plate and placing them into fifteen holes in a wooden board (see Fig. 1). The expert's performance was video-recorded using a high-definition camera, adopting an egocentric perspective to maximize a potential motor resonance effect^35^. The obtained video was used as stimuli for the action observation training (AOT). In addition, during the expert's execution, surface EMG signals were recorded from three hand muscles, namely Opponens Pollicis (OP), First Digital Interosseous (FDI), and Abductor Digiti Minimi (ADM). The choice of the muscles was driven by previous studies^36^ combined with the observation of the natural movement of our model.

During the experimental sessions, participants sat comfortably in front of a 17-inch LCD computer monitor (1024 × 768 pixels) placed 60 cm from their frontal plane and were asked to learn the chopsticks task previously performed by the expert. The training was characterized by six consecutive sessions (S1-S6), each composed of an observation period and an execution one. During observation, subjects were asked to keep their upper limbs relaxed and observe the visual stimuli presented using PsychToolbox-3^37–39^. The AOT participants observed the video (duration 1:04 min) showing the expert performing the task, while the controls observed a video depicting a landscape without any biological movements (same duration). During execution, participants were required to repeat the same motor task performed by the expert, with the middle point of the board chosen as the starting position. The execution time was defined as the interval between the first hand movement and the repositioning of the last marble. Participants had a maximum of 3 min to complete the task. The six executions of each subject were video-recorded from two cameras (lateral and top-frontal views), synched with the simultaneous surface EMG recording.

### Data recording and analysis

According to the video, the reach-to-place of each marble was segmented into three phases: Reaching (from the initial position to the first chopstick-marble contact), Holding (from the successful chopstick-marble contact to the lifting from the plate surface), Transport (from the marble lifting to its positioning in the hole). The duration of the three phases was calculated for each marble and then averaged in each session.

For each execution session, we thus evaluated the following behavioral outcomes:

1. The number of grasping attempts (GA), i.e., the number of contacts between the chopsticks and the marble during the attempt to grab it. In principle, the ideal execution would comprise a number of GA equal to the number of marbles. Conversely, the higher is GA, the more inaccurate the motor performance. This variable has been selected as the primary outcome because the marble grasping is the most challenging phase of the whole task due to the inexperienced participants and the shape/smoothness of the marbles and container;
2. The number of failed liftings (FL), i.e., the number of accidental fallings of the marble during the transport phase, thus impeding its correct positioning on the board;
3. The mean duration of the reach-to-place action (MD) (s), obtained by summing the mean duration of the three phases. In this way, we excluded the time spent failing to grasp from the mean duration, thus obtaining a temporal index completely independent from GA.

As the experimental procedures encompassed the recording of EMG from three hand muscles, subjects were required to produce the maximal contraction of each muscle in separate blocks of about 10 s. These indices were later used for EMG amplitude normalization across participants.

During the task execution, surface EMG signals were recorded from three muscles of the right hand using a wireless EMG system (Cometa Wave Plus, Cometa Srl, Italy). The EMG signals were amplified (× 1000), sampled at 2000 Hz, and filtered with an online first-order band-pass filter (10–500 Hz). The EMG signals were analyzed using a homemade code developed in MATLAB (R2021a) to compute the mean muscular contraction amplitude during the entire reach-to-place action (full trial). For each execution, the amount of muscular contraction was normalized according to the individual (participant and muscle) maximal contraction.

While the parameter described above indexes only the amplitude of the muscular contraction, a point of interest also regards the temporal dynamics of the EMG signal. Thus, the EMG signals were enveloped (rectified and filtered using a band-pass filter 3–1000 Hz and an envelope lowpass 2 Hz filter) and segmented using the same time points extracted from the videos. Subsequently, the EMG signal of each phase was standardized in time (on a temporal axis 1–100). Through this process, we obtained a curve for each phase (reaching, holding, and transport) and muscle, matched in duration and thus comparable in terms of the temporal distribution of the muscular activity. Finally, the curves of the three phases were also concatenated to create a unique curve (full trial). The same procedure was also carried out for the EMG traces of the expert.

The participant's curves were compared with the corresponding ones of the model, adopting the Linear Fit Method (LFM^40^) already used to assess the kinematics similarity in upper limb reach-to-grasp actions^41^ and EMG signals during gait task^42^. LFM calculates the linear regression between the subject's and the model's curves, returning the coefficient R^2^ as a measure of the trueness of the linear relation between them, indicating the temporal similarity between the two curves. When the curves follow the same pattern, the value of R^2^ tends to the ideal value of 1.

In summary, we extrapolated two different EMG information from the participant's training: (1) whether the average contraction amplitude changed over time, and (2) whether the EMG similarity (R^2^) between the trainee and the model changed over time. In the case of positive results, we could also estimate whether these features could lead to a larger behavioral improvement.

### Statistical analysis

To ensure the homogeneity between groups regarding age, chopsticks frequency and ability, and hand dexterity, a two-sample t-test was conducted for each variable. In addition, we also tested the balance of the two groups in terms of baseline performance by submitting the S1 scores of GA, FL, and MD to a two-sample t-test.

The performance scores were baseline corrected (subtracting the S1 scores from the performance scores at each session). Subsequently, single-sample t-tests were employed to evaluate whether groups exhibited a significant learning rate (contrasting the baseline-corrected scores at S6 against 0), and two-sample t-tests to evaluate whether the extent of the learning rate was different across the two groups (contrasting between groups the baseline-corrected scores at S6).

Moving to the EMG analysis, two sample t-tests were conducted to ensure that the initial EMG scores did not differ between groups. Furthermore, three Repeated Measures ANOVAs (one for each muscle) were applied on the average contraction amplitude, with Time as a within-subject factor and Group as a between-subjects factor. The similarity was analyzed to evaluate whether our participants approached the muscular pattern of the model during the training. In case of significant effects, direct comparisons were performed within each group via paired t-tests to explore the differences between the initial (S1) and the final (S6) values. As a note, three subjects had instability of one of the EMG electrodes (1 OP in AOT, 1 OP in CTRL, 1 ADM in CTRL) during the recording. Thus, they have not been included in the relative analyses.

Finally, linear regression analyses were performed to evaluate whether the behavioral amelioration could be explained by the EMG parameters found to be modulated over the training. We standardized the behavioral improvement scores to weigh the absolute increase over the average performance using the formula (Δ = (S6 − S1)/(S6 + S1)). As similarity was already expressed as a percentage, it was enough to compute the difference between the initial and final values (Δ = S6 − S1) to obtain a standardized metric of the convergence toward the model.

The linear regression analyses followed three different hypotheses:

- The initial level of similarity could determine the initial behavioral performance. Should this be the case, the similarity would act as a determinant of the current dexterity of the participant;
- The initial level of similarity could determine the behavioral improvement over the training. Should this be the case, the similarity would act as a determinant of the subject's potential for learning;
- The gain in similarity over the training could explain the behavioral improvement over the training. Should this be the case, the degree of convergence toward the model would act as a determinant for the extent of learning.

Considering that the reach-to-place action is composed of different phases (Reaching, Holding, and Transport) and involves different muscles (OP, FDI, and ADM), we repeated the linear regression analysis separately for each phase and muscle, thus weighting the phase- and muscle-specificity in sustaining the behavioral outcome. Given that the number of comparisons here increased to 9, we applied a False Discovery Rate procedure^43^ to account for multiple comparisons. Finally, we also combined all the similarities within a multiple regression model to assess whether the overall muscular convergence explains the behavioral outcome and whether the linear regression results resist after entering all muscles and phases within a multiple regression model.

## Results

The t-tests showed no significant differences between AOT and CTRL for any baseline variables (all *p* > 0.31), indicating that the two groups were homogeneous in terms of age, chopsticks frequency/ability, and hand dexterity (right and left) (see Table 1). Especially the frequency of chopstick use scores confirmed that our participants do not practice more than once a month (see Supplementary Fig. S1). Concerning the experimental task, the two-sample t-tests on the initial GA, FL, and MD scores (S1) returned no significant difference between groups (all *p* > 0.27). (See Table 1).

**Table 1:** Baseline scores.

	Age	Chopsticks frequency use	Chopsticks ability	NHPT right	NHPT left	GA	FL	MD
AOT	M = 26.08 SD = 4.25	M = 2.39 SD = 0.90	M = 2.80 SD = 1.26	M = 18.34 SD = 3.08	M = 20.06 SD = 3.14	M = 51.42 SD = 22.22	M = 1.42 SD = 1.84	M = 3.91 SD = 0.96
CTRL	M = 25.97 SD = 4.84	M = 2.47 SD = 0.84	M = 2.91 SD = 1.25	M = 19.03 SD = 2.58	M = 20.10 SD = 3.12	M = 50.17 SD = 15.11	M = 0.97 SD = 1.56	M = 3.93 SD = 0.96
T-test	t_(70)_ = 0.10 *p* = 0.92	t_(70)_ = -0.40 *p* = 0.69	t_(70)_ = − 0.37 *p* = 0.71	t_(70)_ = − 1.03 *p* = 0.31	t_(70)_ = − 0.06 *p* = 0.95	t_(70)_ = 0.28 *p* = 0.78	t_(70)_ = − 0.08 *p* = 0.94	t_(70)_ = 1.13 *p* = 0.27

Means and standard deviations of age, chopsticks frequency and ability, right and left Nine Hole Peg Test (NHPT) for the AOT and CTRL groups. In addition, performance scores at S1 for the AOT and CTRL groups are reported. The last line reports the t and *p* values of the relative t-tests. GA refers to the Grasping Attempts, FL to the Failed Liftings, and MD to the Mean Duration.

**Figure 2:**
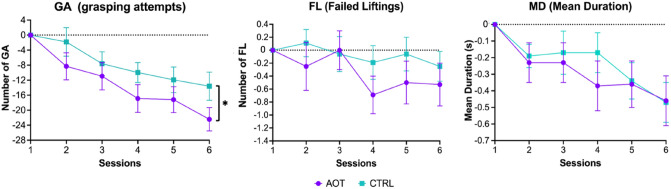
Behavioral results. The graphs indicate how the three behavioral scores develop over time during the six training sessions. Asterisks indicate significant differences in the two-sample t-test (*p* < 0.05). Error bars refer to standard errors.

Figure 2 illustrates the time course of the baseline-corrected behavioral indices for both groups over the six training sessions. As one can see, all of them show a marked performance improvement over the training, with lower values generally found at the final session (S6).

All S6 scores significantly detached from baseline as demonstrated by the results of the one-sample t-tests where both GA and MD returned significant findings [GA: AOT t_(35)_ =  − 7.20, *p* < 0.001; CTRL t_(35)_ = − 4.96, *p* < 0.001], [MD: AOT t_(35)_ =  − 3.00, *p* = 0.005; CTRL t_(35)_ =  − 3.91, *p* < 0.001] while FL exhibited only a trend toward significance but selectively for the AOT group [AOT t_(35)_ =  − 1.61, *p* = 0.12; CTRL t_(35)_ =  − 1.10, *p* = 0.28].

Comparing the two groups in terms of training outcome, a significant difference between AOT and CTRL emerged only for GA[t_(70)_ =  − 2.12, *p* = 0.04, Cohen's d = 0.5] and not for MD and FL [MD: t_(70)_ =  − 0.09, *p* = 0.93; FL: t_(70)_ =  − 0.70, *p* = 0.49].

In summary, the two groups were balanced in their initial performance, both exhibited a significant learning effect over time, but the AOT group outperformed the CTRL one, reflecting a larger decrease in grasping attempts (see Fig. 2).

Moving to the EMG results, the two-sample t-tests on the initial scores (S1) returned no significant difference between AOT and CTRL groups in terms of average contraction amplitude (all *p* > 0.44) and similarity (all *p* > 0.25). Examining the average contraction amplitude over time, the rmANOVA showed no effect of Time or Group for any muscle, suggesting that the extent of muscular contraction is unrelated to behavioral improvement. More interestingly, the same analysis conducted on the trainee-model similarity (R^2^) returned significant or near-to-significance main effect of Time [OP: F(_5,350_) = 2.05, *p* = 0.07; FDI: F(_5,350_) = 3.16, *p* = 0.008] and Time*Group interaction [OP: F(_5,350_) = 2.33, *p* = 0.04; FDI: F(_5,350_) = 1.84, *p* = 0.10] but no significant effect of Group. Conversely, no significant effects were found considering ADM. Overall we can conclude that EMG similarity changes along the training at least for two of the three investigated muscles and that such modulations differ between AOT and CTRL groups.

The paired t-tests conducted within groups between the initial and final R^2^ values revealed a significant difference in the AOT group for both OP and FDI [OP: *M*_S1_ = 0.36, *M*_S6_ = 0.41 t_(34)_ =  − 2.55, *p* = 0.01; FDI: *M*_S1_ = 0.36, *M*_S6_ = 0.42 t_(35)_ = − 2.57 *p* = 0.01], whereas the CTRL group remains virtually unchanged in terms of similarity [OP: *M*_S1_ = 0.36, *M*_S6_ = 0.36, t_(34)_ = 0.09, *p* = 0.93; FDI: *M*_S1_ = 0.39, *M*_S6_ = 0.41, t_(35)_ =  − 1.08 *p* = 0.29].

Summarizing, AOT participants increased their similarity with the model during the training (see Supplementary Fig. S2 for a graphic representation of the EMG pattern convergence toward the model exhibited by an AOT participant). In parallel, the same trend did not appear in CTRL participants, who were not exposed to the model observation. Finally, regression analyses were performed to investigate whether the degree of convergence toward the model could explain the behavioral performance improvement.

Given that GA is the only behavioral outcome showing a significant between-group difference, we used this score as the dependent variable in a linear regression model. The initial level of similarity does not determine the initial behavioral performance (all *p* > 0.48) nor the behavioral improvement over time for both AOT and CTRL groups (all *p* > 0.19). Therefore, a higher initial similarity does not imply better initial dexterity and cannot lead to more behavioral improvement during the training.

We further tested regressions considering the full trial similarity convergence acquired during the training (i.e., the increase of similarity between the first and last training sessions), but no significant results emerged for any muscles in both AOT (all *p* > 0.69) and CTRL (all* p* > 0.16) groups. However, since the three investigated muscles could play different roles in the specific phases of the reach-to-place action, we separately repeated the same analyses considering the Reaching, Holding, and Transport phases. In the CTRL group, no significance emerged for any muscles and phases (all *p* > 0.16), suggesting that the amelioration driven by the mere motor practice is not associated with an increase (and, more generally, a change) of similarity towards an expert model. The multiple regression analysis confirmed such findings, indicating no predictive capacity of similarity on the behavioral improvement (R = 0.50, *p* = 0.53).

**Figure 3:**
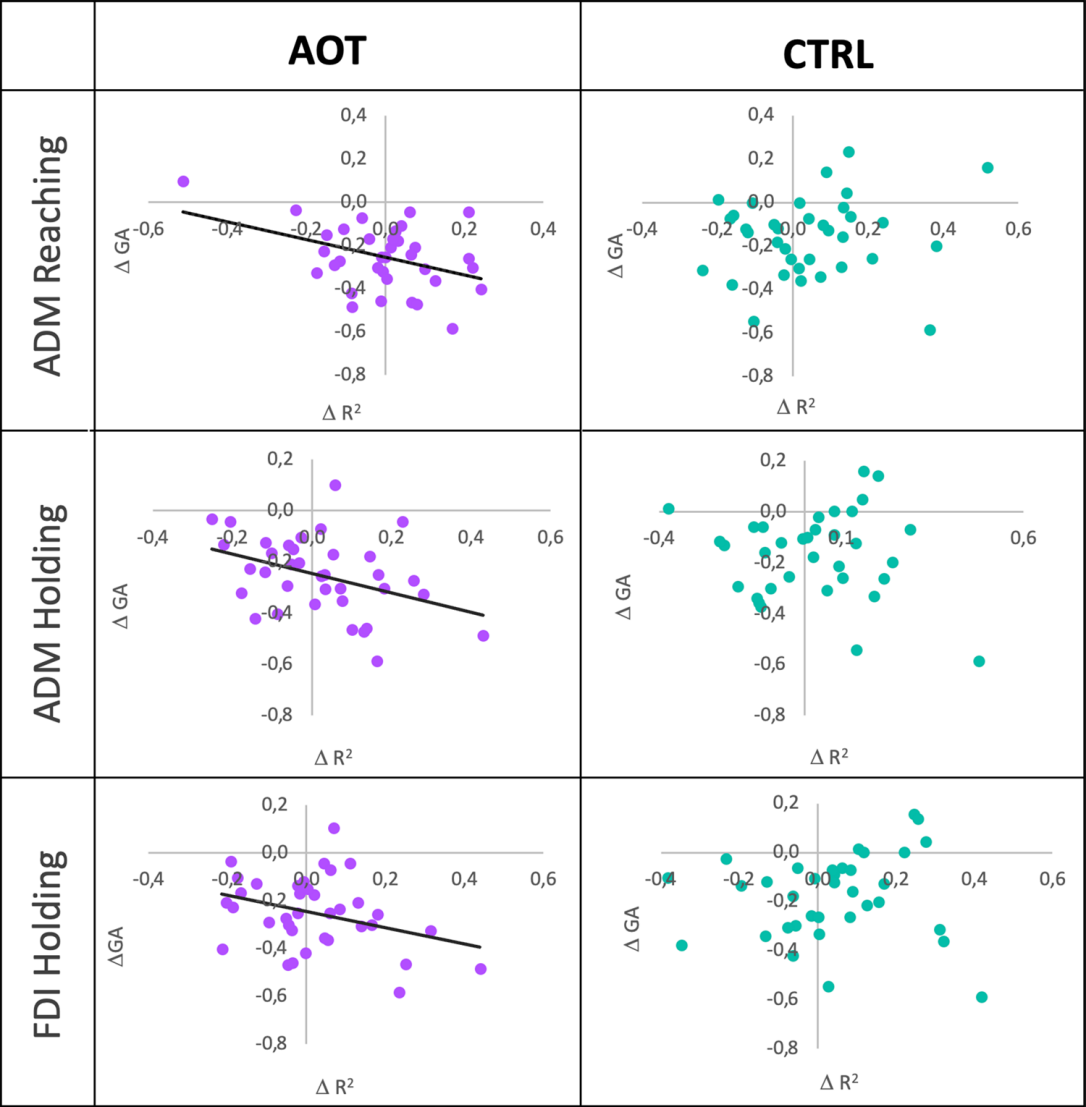
Similarity increases and correlations with behavior. The two columns report the scatterplots about the linear regressions of the GA improvement (delta GA—y-axis) with similarity increase (delta R^2^—x-axis) for the AOT (left column) and CTRL (right column) groups. In case of significant correlations, the trend line is reported in black.

Conversely, several linear regressions appeared significant for the AOT and, in particular, those concerning the gain of similarity of ADM during both the reaching (β = − 0.39, R^2^ = 0.15, *p* = 0.02) and holding (β = − 0.39, R^2^ = 0.15, *p* = 0.02) phases, with FDI during the holding phase showing a trend towards significance (β = − 0.34, R^2^ = 0.11, *p* = 0.04 with threshold after FDR set at 0.02). Not surprisingly, these relationships are negative (i.e., a gain of similarity determines a decrease in grasping attempts) and regard the muscles (FDI and ADM) and temporal phases (reaching and holding) as more involved in grasping with chopsticks (Fig. 3). Of note, these results resist even within a multiple regression model. Indeed, the model accounting for nine variables (the gain of similarity of three muscles per three phases) was almost significant (R = 0.65, *p* = 0.07), with the individual contributions confirming a prominent role of similarity gain for ADM in reaching (β = − 0.41 *p* = 0.02) and holding (β = − 0.40 *p* = 0.06) phases, and only marginal, non-significant, contributions of other variables.

## Discussion

The present study investigated how the muscular activation underlying a complex motor task changes along AOT and whether these modulations parallel the behavioral improvements to some extent. For these purposes, seventy-two healthy subjects were enrolled, randomly sub-divided into two groups (AOT and CTRL), and administered training to learn to grasp marbles with chopsticks.

We observed a significant improvement for all groups in several behavioral indices, with AOT outperforming the CTRL group, especially in the number of grasping attempts, whose decrease was almost double. This finding confirms the results of previous studies^6–10^, suggesting that the alternation between action observation and execution represents an effective strategy to promote the learning of complex motor skills. Indeed, action observation activates the motor system according to the observed motor program (see^1^), whereas, during the subsequent execution, the subject acts with a motor system already pre-activated and biased toward the correct performance^2^.

A relevant aspect of our study is represented by the chosen task, selected as a behavior not belonging to the participants' cultural and motor repertoire. The underlying reasons are threefold: 1) grasping marbles with chopsticks is highly demanding, thus limiting possible ceiling effects during learning; 2) being chopsticks uncommonly used in Western cultures, it would have been easier to recruit a large sample of participants as naïve as possible to the required motor task, further guaranteeing the initial dexterity balance; 3) finally, a previous study^44^ had already demonstrated that naïve participants could learn a chopsticks task via motor practice with a performance improvement following the logarithmic pattern typical of motor learning. The complexity of the selected motor task impeded to derive an overall score. Thus, we extracted multiple quantitative indicators indexing different aspects of the motor performance to render a multifaceted picture of the behavioral performance with scores related to dynamic control of the grip (GA), static maintenance of the grip (FL), and overall speed (MD). AOT turned out to considerably impact the capacity of the participants to control the contact with the marble successfully. At the same time, the choice of chopsticks might have also limited the learning rate. Indeed, some behavioral improvements here (e.g., the static control of the marble grip) are reasonably due to the capacity of subjects to adjust their performance according to internal feedback (e.g., proprioception or goal achievement) experienced during motor practice more than external inputs like action observation. We could then conclude that the behavioral advantage of AOT is mainly confined to the dynamic control of the marble grip, with other aspects somewhat mitigated by the nature of the chosen task.

The analysis of the muscular activity did not reveal any training-related modulation of the contraction amplitude in both group. Previous studies suggested that action observation, in isolation or combined with motor imagery, enhances muscular activity during the execution of the same task^29–31^. However, they all involved force tasks devoid of precise motor control components. Interestingly, one study on dart-throwing indicated a reduction of arm muscle contraction along with performance improvement due to motor training^32^. With this notion in mind, we hypothesized that increasing proficiency in using chopsticks might have been paralleled by a decreasing contraction of the hand muscles during the task, and our results indicated that this is not the case. In the attempt to harmonize the apparently contrasting results between our study and Romano Smith et al.^32^, we can propose that the more complex task adopted in our study could have delayed the temporal effect of the training on EMG contraction amplitude. However, we cannot be conclusive, as demonstrations require longer training for tasks with different complexity levels.

Contrary to the case of contraction amplitude, the similarity between the participants and the model in muscular temporal dynamics significantly changed during the training. Specifically, we revealed a gain in EMG similarity standing only for the AOT (5%) and not for the CTRL group (0%). This result is even more relevant if one considers that both groups presented a significant behavioral improvement during the training, demonstrating that motor learning still happens in the absence of previous action observation but follows trajectories unbiasedly relative to the model. In other words, CTRL participants are free to search their ameliorative strategies, and this freedom does not increase similarity with the expert at the population level. Different instead is the case of AOT, as it exposes subjects to expert observation and rehearses their motor system accordingly, polarizing the learning trajectory towards the model and ultimately explaining why AOT participants significantly increase their similarity over time.

Notably, the similarity gain achieved during the training predicted the behavioral learning rate selectively for AOT participants. Significant regressions regarded only the muscles and action phases surrounding the grasping events, namely FDI and ADM during reaching and holding. Despite further evidence being needed, the notion that during AOT, participants' improvement is driven by the absorption of some motor fingerprints of the model opens different potential applications and uses. For instance, several motor tasks require maximizing the independence between different muscular districts (agonists vs. antagonists) to increase performance and reduce the fatigue and risks of injuries. In such a scenario, AOT could thus play a decisive role in focusing the training on specific muscular districts.

The role that the agent-observer motor similarity exerts on the observer's motor system is not limited to indexing the AOT outcome but also extends to supporting cognitive functions such as the capacity to predict the goal of the observed action^41^. These aspects indicate how the model-observer motor similarity represents a feature that needs to be carefully designed (and adjusted over time) within action observation training procedures according to the individual trainee characteristics and the scopes of the training.

Control analyses ruled out an effect of the initial level of similarity on both the behavioral performance at S1 and the behavioral improvement during the training. These findings may sound in contrast with those by De Marco et al.^41^, as one could postulate that the better intention prediction accompanying the observation of a highly similar action should ground on a stronger motor responsiveness to that action observation. However, it is reasonable to assume that this scenario stands mainly when the observed actions somewhat belong to the observer's motor repertoire, i.e., when he/she has acquired some degree of familiarity. This is not the case in our study, as subjects were randomly selected as naïve to the trained task.

**Figure 4:**
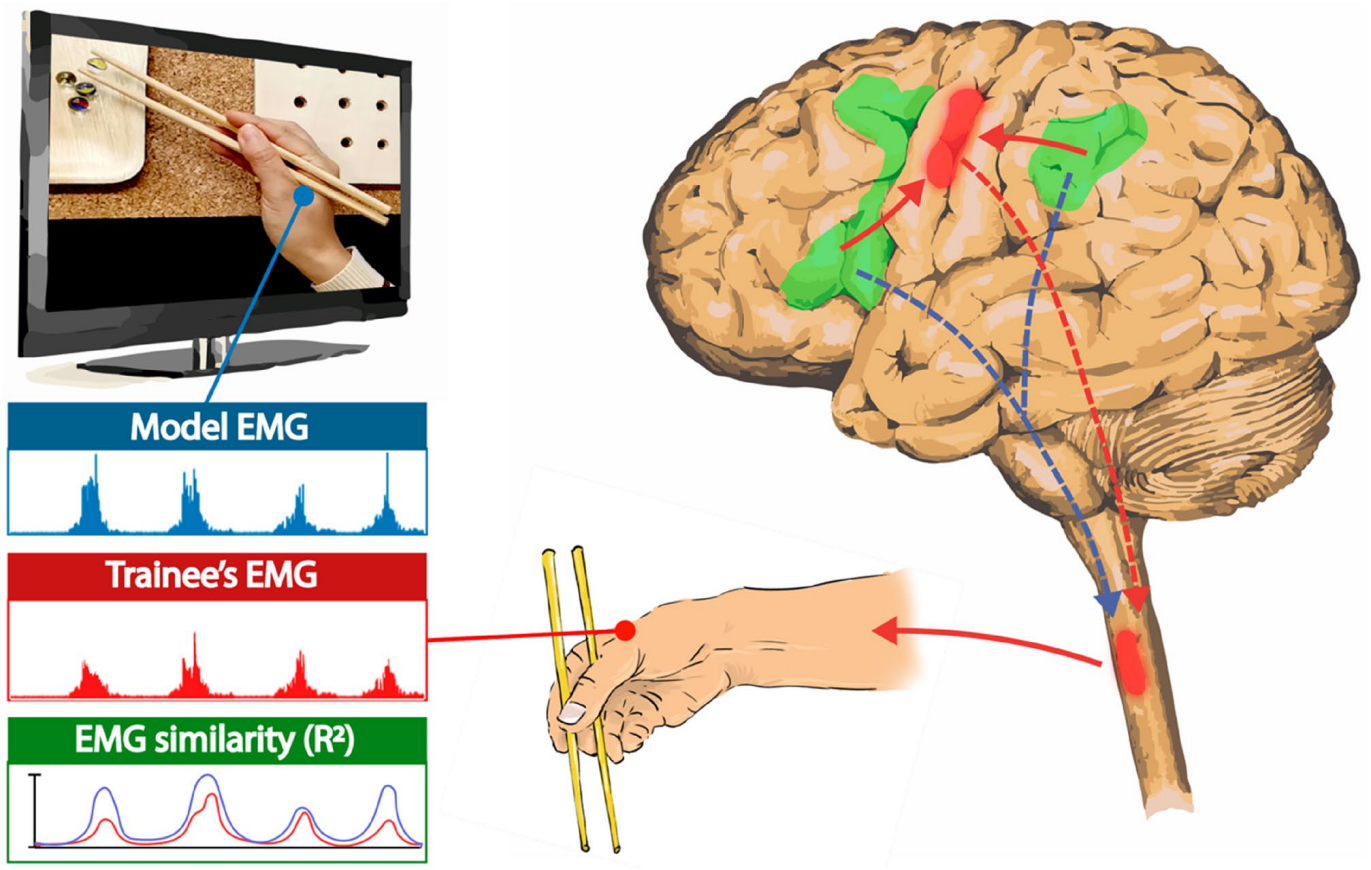
Model explaining the AOT effect on behavioral improvement and the similarity convergence toward the model. Green areas represent frontal and parietal areas endowed with the mirror mechanism. Their projections to the primary motor cortex (red area) are highlighted with continuous red arrows; corticospinal projections (from premotor, parietal, and primary motor cortices) are represented with dashed arrows.

Considering the organization of the motor system, two non-mutually exclusive models might explain the role of action observation in promoting the convergence toward the model and consequently leading to larger behavioral improvement (see Fig. 4). First, cortico-cortical projections from premotor^45^ and parietal^46^ areas, cortical hubs of the mirror mechanism, may activate the primary motor cortex (M1), forging the motor representations of the spatiotemporal features involved in the complex task. Second, direct^47^ descending corticospinal projections from parietal and premotor regions^48^ via disynaptic outputs^47,49–55^ could impact the spinal excitability, thus inducing spinal plastic changes underlying the hand's motor control improvement^56,57^.

This work is not without its limitations. Our sample is unbalanced in terms of participants' gender (76.39% of participants are female). Such unbalance might bias the results of our study, considering that males and females have different susceptibility to learning motor skills^58^, and brains under different sexes may have different salience or gene expression values in different brain regions^59–61^. While such a bias might affect the overall learning curves, it must be highlighted that we preserved an identical gender balance across the two groups, with AOT composed of 28 females—8 males and CTRL 27 females—9 males. Thus, we can reasonably rule out that the prevalence of a specific gender drives the reported differences between groups. Nevertheless, further studies are needed to test the reliability of our findings across genders, possibly making parallelisms with the different reactivity of the mirror mechanism between males and females^62^.

Another limitation derives from the fact that AOT impacted only some aspects of motor behavior, and the increase of similarity regards only a few combinations of muscles/phases significantly associated with behavioral improvement. As mentioned above, the complexity and specificity of our task could have mitigated the potential impact of AOT, de facto underpowering the effect of visual feedback on motor components relying mainly on proprioceptive control. However, the paucity of significant improvements can also be seen as proof of the specificity of the AOT effect, which is limited to the motor act involving a dynamic control of the movement (i.e., grasping of the marble) and to the combinations of muscles/phases mostly related to the same gesture. What remains is the need to generalize our findings employing different motor tasks, thus assessing precisely which aspects of complex motor behaviors can be targeted via AOT and which ones can be monitored via an online assessment of the EMG similarity.

Finally, we must acknowledge that the effect size of the behavior-similarity link is modest, with an explained variance of around 15% for linear regression models. Here, the simplicity of our behavioral tracking and the complexity of the administered task might have tempered these associations. Future studies employing simpler tasks as well as more sensitive behavioral outcomes (e.g., kinematic tracking) might help refine the strength of this relationship, further informing about the possibility of predicting the learning rate via an online similarity assessment.

## Conclusions

In the present study, we demonstrated that the amelioration of the motor performance induced by AOT parallels the degree of convergence of the motor pattern of the observer toward that exhibited by the model, even at the muscular, peripheral level. The significance of these results is twofold: first, they witness that motor resonance, supporting the efficacy of AOT at the cortical level^28^, also reflects at the muscular level, i.e., one step nearer to the behavior along the descending motor chain. From these premises, we could envision monitoring the motor similarity with the model over the training, thus deriving correlates of the instantaneous AOT efficacy and valuable, poorly invasive inputs for neurofeedback protocols. Innovative hardware and software solutions could further facilitate this scenario. While the former regard wearable, wireless sensors ensuring ecological tracking of electrophysiological data during daily activities and tasks^63^, the latter relate to advanced computational and mathematical models that might help in refining, tailoring, and optimizing the extraction of electrophysiological features such as the EMG similarity^64–67^.

## Supplementary Information

Supplementary Figures.

## Data Availability

The data that support the findings of this study are available from the corresponding author upon reasonable request.
