# Supplementary material for: The capacity of action observation to drag the trainees' motor pattern toward the observed model
Source: Sci Rep. 2023 Jun 5;13:9107. doi: 10.1038/s41598-023-35664-w (PMC10241915; doi:10.1038/s41598-023-35664-w)
Supplement: Supplementary file 1 — Supplementary Figures. [file 41598_2023_35664_MOESM1_ESM.docx]

**Supplementary Material**

1. **Distribution of the questionnaire scores within the two groups.**


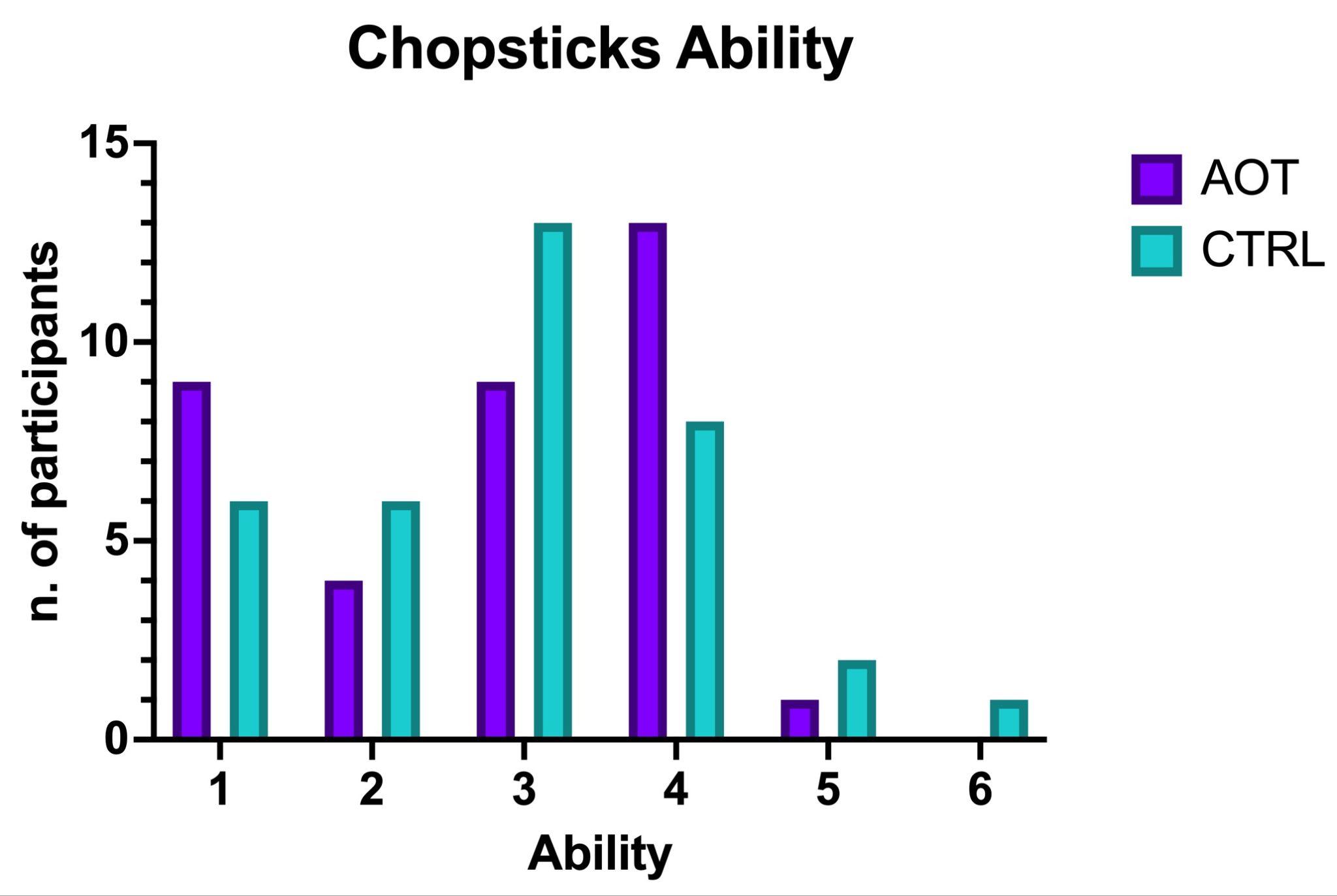

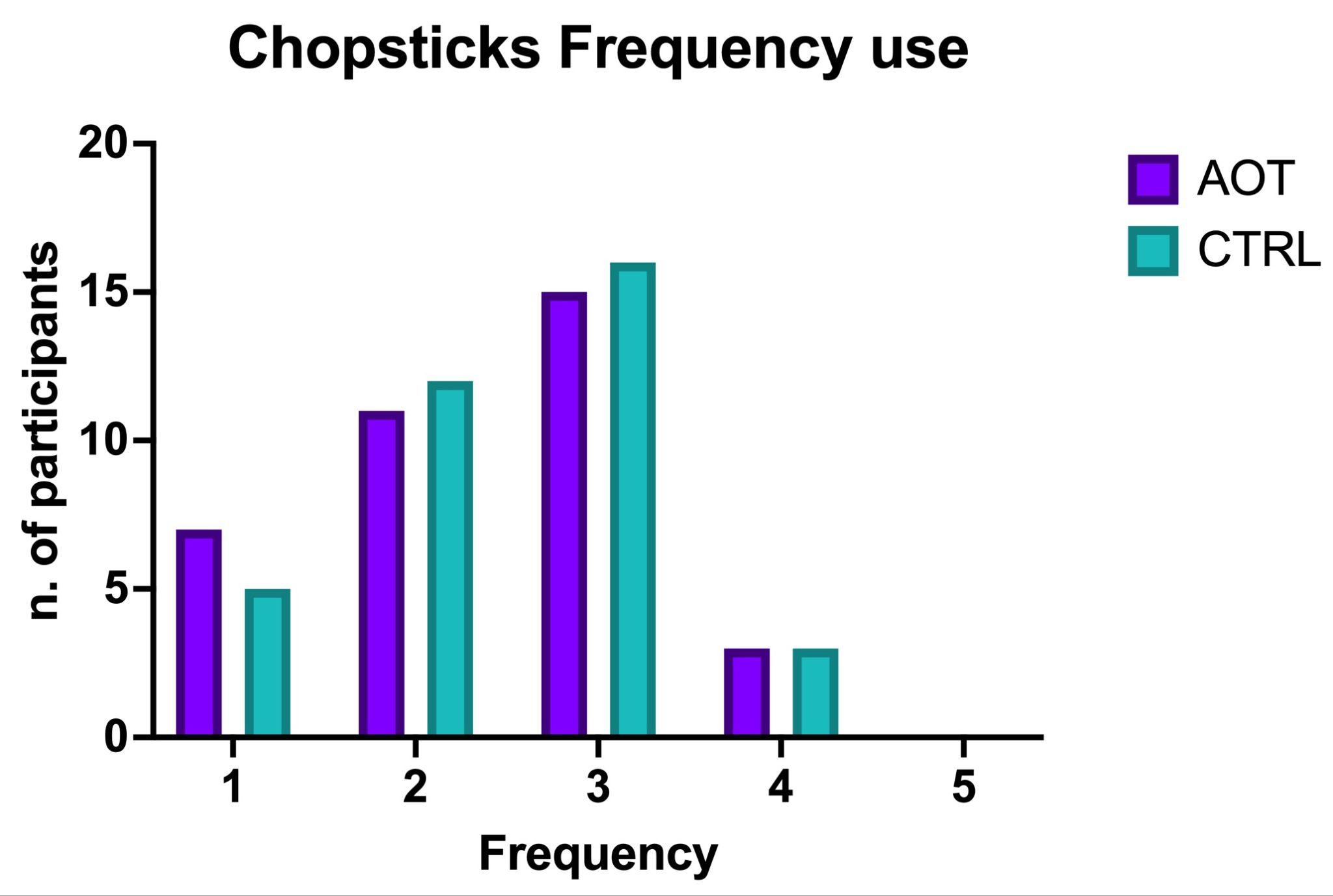


***Figure S1.*** *These graphs represent the distributions across the two groups in terms of expertise with chopsticks; the left panel shows the distribution of the self-reported ability with chopsticks (rating 1-6), and the right panel shows the distribution of chopsticks frequency use (rating 1-5).*

**2. Convergence toward the model, in terms of EMG temporal dynamics, from the initial to the final training session.**


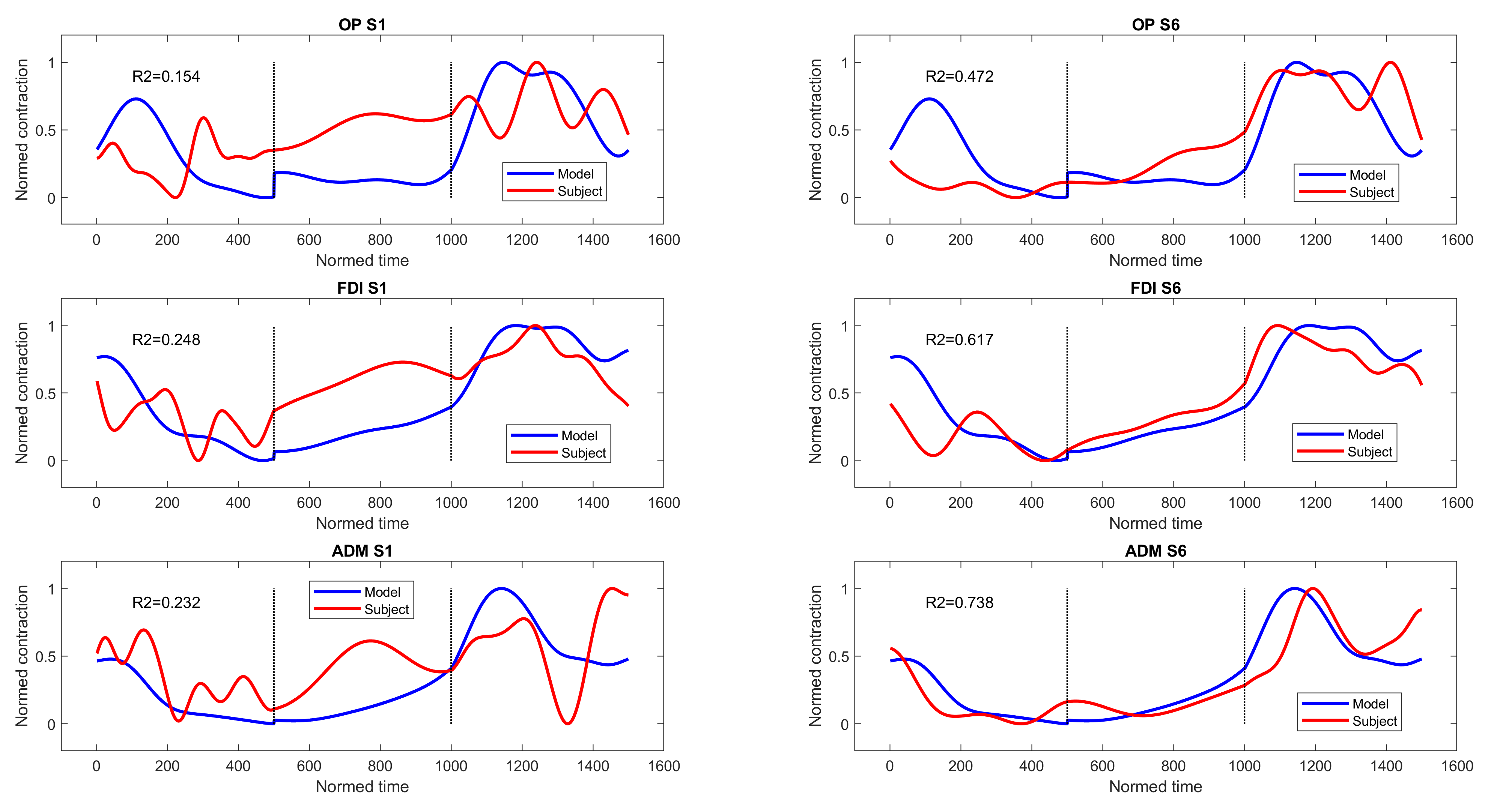


***Figure S2.*** *Mean EMG time courses of the model (blue lines) and a representative AOT participant (red lines) during the first (S1) and the last (S6) training sessions. The vertical dotted lines within each panel delimited the three phases of the action (reaching, holding, and transport). The rows relate to the three muscles registered during the training (OP, FDI, ADM). X-axis indicates the normalized time (500 time-points for each phase), Y-axis indicates the normalized EMG amplitude after rescaling between 0 and 1.*
